# The association between oral health status and physical function in elderly patients with acute heart failure

**DOI:** 10.1002/cre2.824

**Published:** 2023-12-17

**Authors:** Yusuke Uemura, Rei Shibata, Shinji Ishikawa, Kenji Takemoto, Toyoaki Murohara, Masato Watarai

**Affiliations:** ^1^ Anjo Kosei Hospital Cardiovascular Center Anjo Japan; ^2^ Department of Advanced Cardiovascular Therapeutics Nagoya University Graduate School of Medicine Nagoya Japan; ^3^ Department of Cardiology Nagoya University Graduate School of Medicine Nagoya Japan

**Keywords:** activities of daily living, acute heart failure, elderly patients, oral health

## Abstract

**Objectives:**

Oral health problems are common and are associated with various geriatric conditions in older adults. The importance of oral health has not been fully highlighted in the assessment and management of patients with heart failure. Here, we investigated the association between oral health status and the decline in physical function during hospitalization in elderly patients with acute heart failure.

**Materials and Methods:**

We evaluated oral health using the revised oral assessment guide in 77 patients aged 65 years or older who were admitted to hospital for acute heart failure. Oral health problems were defined as a revised oral assessment guide score ≥9.

**Results:**

Oral health problems were identified in 66.2% of the patients. Patients with oral health problems had high prevalence of decreased physical function, undernutrition, and cognitive impairment. A reduction in the Barthel Index, as an indicator of activities of daily living during hospitalization, was significant in the enrolled patients. The Barthel Index decreased more in patients with oral health problems than those with normal oral health. Furthermore, the revised oral assessment guide score on admission was found to be the only independent predictor of changes in the Barthel Index during hospitalization in the multivariate analyses.

**Conclusions:**

Oral assessment using the revised oral assessment guide during hospitalization could provide useful information for the management of elderly heart failure patients.

## 1 INTRODUCTION

The incidence and prevalence of heart failure (HF) increases with age (Benjamin et al., 2018; Bui et al., 2011; Conrad et al., 2018; Shimokawa et al., 2015). HF is the most common cause of hospitalization in patients aged 65 years or older. Hospitalization for HF is a significant public health problem, especially in elderly patients, because it is associated with higher rates of mortality, repeated rehospitalization, and decline in physical activity, compared to that in younger patients (Chioncel et al., 2017; Hamaguchi et al., 2010; Lawson et al., 2019; Uemura et al., 2018).

Elderly HF patients generally have complex comorbidity profiles, including not only comorbid diseases, but also geriatric conditions (Afilalo et al., 2014; Conrad et al., 2018; Henkel et al., 2008). These complex and diverse profiles are associated with poor prognosis of HF and increase the burden on healthcare services after discharge due to the decline in physical function and activities of daily lives (ADL) (Chaudhry et al., 2010; van Deursen et al., 2014). Thus, a multidisciplinary approach, as well as medical treatment, is becoming important to preserve patients’ ability to perform ADL during hospitalization are important in the management of acute HF.

Oral health problems, like dry mouth, periodontal disease, dental caries, and inappropriate dentures, are common health conditions in older adults (Kassebaum et al., 2017; Marcenes et al., 2013). Previous studies have demonstrated the association between poor oral health and various geriatric conditions, such as frailty, undernutrition, cognitive impairment, and decline in physical function (Azzolino et al., 2019; Hasegawa et al., 2019; Kossioni, 2018; Ramsay et al., 2018; Shiraishi et al., 2019). However, the importance of oral health care has not been fully highlighted in patients with HF. In the present study, we investigated the association between oral health status and the decline in physical function during hospitalization in elderly patients with acute HF.

## 2 MATERIALS AND METHODS

### 2.1 Study population

This is a single‐center retrospective cohort study. We reviewed 171 consecutive patients who were admitted for the treatment of acute HF between October 2018 and March 2019. All patients were diagnosed with HF using the Framingham criteria (McKee et al., 1971). Briefly, the Framingham criteria for the diagnosis of HF are based on symptoms and findings of left HF, right HF, and low cardiac output. It includes eight major criteria (acute pulmonary edema, cardiomegaly, hepatojugular reflux, neck vein distention, orthopnea, pulmonary rales, third heart sound, and weight loss >4.5 kg in 5 days in response to treatment) and six minor ones (ankle edema, dyspnea on exertion, hepatomegaly, nocturnal cough, pleural effusion, and tachycardia with >120 beats per minute). HF is diagnosed with two or more major criteria, or one major criterion plus two minor criteria.

During hospitalization, treatment for acute HF was provided for all the patients based on the Japanese guidelines of acute and chronic HF, including adequate oxygenation, pharmacological and nonpharmacological therapy, and management of underlying disease (Tsutsui et al., 2019). Furthermore, standardized cardiac inpatient rehabilitation was provided for all the patients according to standardized cardiac rehabilitation program from the Japanese Association of Cardiac Rehabilitation (Izawa et al., 2019).

The study was approved by the Anjo Kosei Hospital ethics committee (Approval No. R19‐032). Because of its retrospective nature, informed consent was deemed unnecessary according to the national regulation issued by the Japanese Ministry of Health, Labour and Welfare. However, the present study was carried out by the opt‐out method of our hospital website.

A medical history was obtained to document past medical history, medications, and co‐morbid disease. Hypertension was defined as systolic blood pressure (BP) ≥ 140 mmHg or diastolic BP ≥ 90 mmHg on repeated measurements, or receipt of antihypertensive treatment. Diabetes mellitus was defined as having a blood hemoglobin A1c ≥ 6.5%, 2‐h value ≥200 mg/dL (≥11.1 mmol/L) on a 75 g oral glucose tolerance test, and/or taking glucose‐modulating medication according to the diagnostic criteria of the Japan Diabetes Society (Seino et al., 2010).

### 2.2 Oral assessment

Oral health was assessed by a certified dysphagia nurse using the revised oral assessment guide (ROAG) (Andersson et al., 2002). The ROAG score was used in a previous study that investigated its association with clinical outcomes in post‐acute care settings (Shiraishi et al., 2019). The reliability and validity of the ROAG have been reported (Konradsen et al., 2014; Ribeiro et al., 2014). ROAG includes eight categories: voice, lips, mucous membranes, tongue, gums, teeth/dentures, saliva, and swallowing. Each category was described and rated as healthy (Score 1) to severe (Score 3). The total score ranged from eight, representing a normal oral health, to 24, which represents severe oral health problems. The ROAG score was obtained within 1 week of hospitalization when the patient's respiratory state was stabilized without the need for oxygen. In the present study, patients with ROAG scores of 8 points were regarded to have a normal oral health and those with higher scores were regarded as having poor oral health (mild to moderate oral problems: 9–12 points, severe oral problems: 13–24 points).

### 2.3 Measurements of geriatric conditions

Physical functional status was evaluated using the Barthel Index (BI), handgrip, and 10‐m normal/usual gait speed. The BI was obtained by ward nurses at admission and at discharge, as previously reported (Uemura et al., 2018). Changes in the BI were calculated as the difference between the BI on admission and the BI on discharge. Handgrip and 10‐m gait speed were evaluated by physical therapists before discharge.

Registered dieticians assessed nutritional status. Nutritional status was screened using the controlling nutritional status (CONUT) score and the geriatric nutritional risk index (GNRI) (Bouillanne et al., 2005; Ignacio de Ulíbarri et al., 2005). Laboratory data at admission and body mass index (BMI) at the first measurement within 72 h of hospitalization were used for calculation of the scores. Dietary energy intake was assessed by the proportion of nutritional intake from food compared to the predicted calorie requirement. Nutritional intake was calculated based on the food intake for 3 days around the day of oral assessment. The predicted calorie requirement was defined as the total energy expenditure estimated from the Harris–Benedict equation (Harris & Benedict, 1918).

Pharmacists assessed cognitive function using the mini‐mental state examination (MMSE) before discharge (Folstein et al., 1975).

### 2.4 Biomarker analysis and echocardiography

Blood samples were obtained at the time of hospital admission. Complete blood counts were performed utilizing a Sysmex XE‐5000 analyzer (Sysmex). Plasma BNP was measured with the AIA‐2000 enzymatic immunoassay analyzer (TOSOH). Other biomarkers were measured using a LABOSPECT 008 autoanalyzer (Hitachi Co.). The estimated glomerular filtration rate (eGFR) was calculated by the Modification of Diet in Renal Disease formula (Matsuo et al., 2009). Echocardiographic examination was performed by an experienced sonographer using Vivid E9 with XD clear (GE Healthcare). The images were recorded in a console and analyzed offline. Left ventricular ejection fraction was calculated using the modified Simpson's rule.

### 2.5 Outcomes

A previous study demonstrated that patients whose BI score decreased by ≥20 have poor prognosis (Katano et al., 2021; Obata et al., 2021). Therefore, we defined a decrease in BI score by ≥20 during hospitalization as the primary outcome. The association between a decrease in BI during hospitalization and ROAG score was also examined.

### 2.6 Sample size calculation

We defined a decrease in BI score by ≥20 during hospitalization as the primary outcome. Furthermore, in our previous data, the BI scores of patients admitted for the treatment of acute HF were normally distributed, with a standard deviation of approximately 20 (Uemura et al., 2018). If the true difference in means between patients with and without oral health problems is 20, a sample size of 16 would be needed in each group to reject the null hypothesis with a power of 0.8 and an alpha error of .05. Accordingly, in the present study, data of a minimum of 18 patients were obtained in each group. Assuming a 10.0% loss due to in‐hospital death, we aimed to enroll 40 patients.

### 2.7 Statistical analysis

All analyses were performed using PASW Statistics 21 software (SPSS Inc.). Continuous variables were presented as the mean ± standard deviation or median (interquartile range). We assessed the normal distribution of continuous variables by the Shapiro–Wilk test. One‐way analysis of variance (ANOVA) or Kruskal–Wallis test was used to determine the mean or median differences in variables between the groups. Categorical variables were presented as the count and/or percentage. Categorical variables were presented as the count and/or percentage and the chi‐square test or Fisher's exact test was used for the group comparisons. To identify the predictors of BI decrease by ≥20 during hospitalization, univariate and stepwise multivariate logistic regression analyses were used to calculate the odds ratios and 95% confidence interval. Univariate correlations between changes in the BI during hospitalization and other variables were investigated using Pearson's rank correlation test, and then a stepwise multiple linear regression analysis was performed. Variables with *p* < .1 in the patients’ characteristics were selected as covariates to adjust for bias. Furthermore, body mass index, history of HF admission, and BI at admission were also selected, because these have been reported to be clinically relevant for decreased BI during hospitalization in patients with HF (Itoh et al., 2020; Saitoh et al., 2021). Age, gender, and variables with *p* < .05 in the univariate analyses were incorporated into the multivariable model. In all analyses, *p* < .05 was considered statistically significant.

## 3 RESULTS

**Figure 1:**
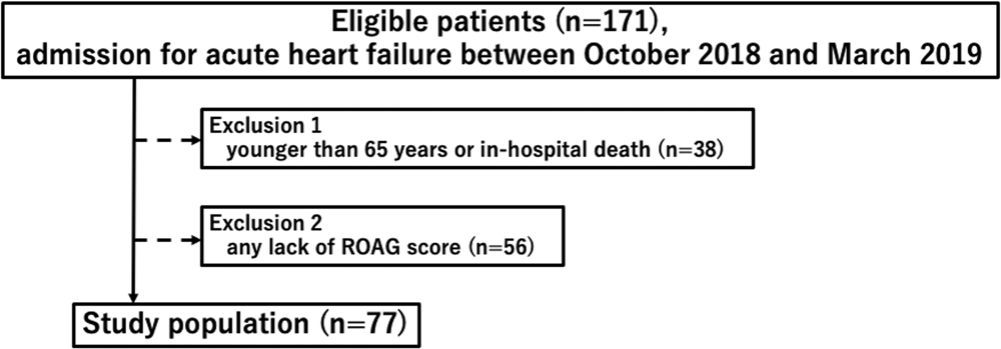
Flow chart of the study population. ROAG, revised oral assessment guide.

Among a total of 171 eligible patients, 38 patients younger than 65 years or those with in‐hospital death were excluded. We also excluded 56 patients whose ROAG score could not be obtained. Finally, the study included 77 patients (Figure 1). The mean age of the overall patients was 80.0 ± 9.1 years, and 58.4% of patients were men. The mean ROAG score was 9.9 ± 2.2. Patients with oral health problem (ROAG score ≥9) were identified in 66.2% of the enrolled patients. Details of ROAG evaluations are shown in Table 1.

**Table 1:** Details of ROAG evaluation of the enrolled patients.

Items	Evaluation (score)		
	Normal (1)	Minor problems (2)	Severe problems (3)
Voice	47	27	3
Lips	41	36	0
Mucous membranes	75	2	0
Tongue	60	17	0
Gums	73	4	0
Teeth/dentures	62	13	2
Saliva	58	11	8
Swallowing	65	10	2

*Note*: Data are presented as the number of patients.

Abbreviation: ROAG, revised oral assessment guide.

Table 2 shows patients’ baseline characteristics. Patients with oral health problems were older and had lower albumin levels. C‐reactive protein levels were higher in patients with oral health problems than in those with normal oral health. History of stroke was highly prevalent in patients with oral health problems. In measurements of geriatric conditions, handgrip strength, GNRI, dietary energy intake, and MMSE were significantly lower in patients with oral health problems than in those with normal oral health. The COUNT score was significantly higher in patients with oral health problems than those with normal oral health.

**Table 2:** Baseline characteristics of enrolled patients according to ROAG score‐evaluated oral health status.

	Normal (*n* = 26)	With oral problems (*n* = 51)	*p* Value
ROAG score	8	10.9 ± 2.1	<.001
Age (years)	73.9 ± 8.3	83.1 ± 7.9	<.001
Male sex	19 (73.1%)	26 (51.0%)	.063
Body mass index (kg/m^2^)	23.1 ± 4.6	21.7 ± 3.5	.159
History of admission due to HF	14 (53.8%)	21 (41.2%)	.291
History of CAD	8 (30.8%)	18 (35.3%)	.691
History of stroke	3 (11.5%)	8 (15.7%)	.623
Current smoking	2 (7.7%)	2 (3.9%)	.481
Hypertension	21 (80.8%)	44 (86.2%)	.529
Diabetes mellitus	7 (26.9%)	22 (42.3%)	.165
Atrial fibrillation	13 (50.0%)	23 (45.1%)	.683
Hemoglobin (g/dL)	12.1 ± 2.7	11.1 ± 2.0	.071
CRP (mg/dL)	0.25 (0.13–0.86)	1.24 (0.28–4.19)	.009
Albumin (g/dL)	3.6 ± 0.5	3.3 ± 0.5	.014
Creatinine (mg/dL)	1.69 ± 1.20	1.55 ± 0.92	.580
eGFR (mL/min/1.73 m^2^)	42.5 ± 22.7	38.7 ± 19.5	.446
Sodium (mEq/L)	139.0 ± 3.7	140.3 ± 3.4	.128
BNP (pg/mL)	992.0 (415.5–1687.2)	732.8 (531.9–1101.0)	.598
LV ejection fraction (%)	39.7 ± 16.6	46.5 ± 17.5	.106
Concomitant respiratory infection	1 (3.8%)	14 (27.5%)	.013
Use of NIPPV at admission	4 (15.4%)	11 (21.6%)	.517
Use of inotropes	4 (15.4%)	7 (13.7%)	.844
Length of bed rest (days)	2 (1–3)	3 (2–5)	.059
Medications on admission			
ACE inhibitors/ARBs	12 (46.1%)	19 (37.3%)	.451
Beta‐blockers	16 (61.5%)	25 (49.0%)	.298
Calcium channel blockers	11 (42.3%)	21 (41.2%)	.924
Loop diuretics	16 (61.5%)	30 (58.8%)	.818
MRAs	6 (23.1%)	7 (13.7%)	.300
Tolvaptans	8 (30.8%)	14 (27.5%)	.761
Measurements of geriatric conditions			
Handgrip strength at discharge (kg)	20.2 ± 8.7	14.5 ± 6.6	.040
Gait speed for 10‐m walk at discharge (s)	14.8 ± 6.2	19.5 ± 7.5	.069
CONUT score at admission	4.3 ± 2.9	5.7 ± 2.8	.045
GNRI at admission	98.1 ± 14.3	87.0 ± 9.9	<.001
Use of dentures at admission	14 (53.8%)	33 (64.7%)	.355
Dietary energy intake at discharge (%)	89.6 ± 18.7	67.5 ± 32.3	.002
MMSE at discharge	27.4 ± 2.4	25.2 ± 3.4	.047

*Note*: Data are presented as mean ± standard deviation (SD), median (interquartile range [IQR]), or *n* (%).

Abbreviations: ACE, angiotensin‐converting enzyme; ARBs, angiotensin receptor blockers; BNP, brain natriuretic peptide; CAD, coronary artery disease; CRP, C‐reactive protein; eGFR, estimated glomerular filtration rate; HF, heart failure; LV, left ventricular; MRA, mineralocorticoid receptor antagonists; NIPPV, noninvasive positive pressure ventilation; ROAG, revised oral assessment guide.

The actual BI values on admission and discharge, and the BI changes in each group are shown in Table 3. Reduction in the BI during hospitalization was significant in the enrolled patients in both the normal oral health group and oral health problems group (*p* < .01). Of note, the BI decreased more in patients with oral health problems than those with normal oral health (*p* < .01). (Table 3).

**Table 3:** Changes in the Barthel index during hospitalization.

	Normal (*n* = 26)	With oral problems (*n* = 51)	*p* Value
Barthel Index at admission	96.0 ± 9.8	76.6 ± 30.8	.004
Barthel Index at discharge	93.5 ± 12.8	61.7 ± 39.5	<.001
Changes in Barthel Index	−1.2 ± 6.6	−11.0 ± 21.5	.036
Length of hospital stay (days)	11 (8–16)	12 (8–19)	.654

*Note*: Data are presented as mean ± standard deviation (SD) or median (interquartile range [IQR]).

Tables 4 and 5 show the results of the multivariate analyses for decline in BI during hospitalization adjusting for potential covariates. Multiple logistic regression analysis revealed that the ROAG score at admission was independently associated with BI decline by ≥20 (odds ratio: 1.497, 95% confidence interval: 1.077–2.082; *p* = .016) (Table 4). Furthermore, changes in the BI during hospitalization were significantly correlated with age, hemoglobin levels, ROAG score, GNRI, and dietary energy intake on admission in the univariate regression analysis. Of those, ROAG score on admission was the only independent predictor of changes in the BI during hospitalization in the multivariate regression analyses (Table 5).

**Table 4:** Logistic regression analyses for BI decrease by ≥20.

	Univariate analysis			Multivariate analysis		
	OR	95% CI	*p* Value	OR	95% CI	*p* Value
Age	1.124	1.033–1.224	.007	–	–	–
Male sex	1.543	0.472–5.047	.473	–	–	–
Body mass index	0.938	0.742–1.186	.595			
History of admission due to HF	0.759	0.241–2.389	.637			
Hemoglobin	0.813	0.623–1.062	.129			
Log CRP	2.120	0.853–5.267	.106			
Albumin	0.341	0.107–1.086	.069			
Concomitant respiratory infection	1.042	0.253–4.281	.955			
Log length of bed rest	5.385	0.922–31.438	.061			
ROAG score	1.653	1.219–2.240	.001	1.497	1.077–2.082	.016
Barthel Index at admission	0.992	0.973–1.011	.408			
Handgrip strength	0.871	0.698–1.086	.221			
Gait speed for 10‐m walk	1.118	0.921–1.356	.259			
CONUT score	1.444	1.150–1.812	.002	1.329	1.039–1.700	.023
GNRI	0.921	0.861–0.986	.019			
Dietary energy intake	0.975	0.957–0.993	.007	–	–	–
MMSE	0.855	0.610–1.199	.365			

Abbreviations: CI, confidence interval; CONUT, controlling nutritional status; CRP, C‐reactive protein; GNRI, geriatric nutritional risk index; HF, heart failure; MMSE, mini‐mental state examination; OR, odds ratio; ROAG, revised oral assessment guide.

**Table 5:** Independent predictors of change in the Barthel index based on linear regression analyses.

	Univariate		Multivariate				
	*β*	*p* Value	*β*	*p* Value	*B*	SE	95% CI
Age	−.357	.004	–	–	–	–	–
Male sex	−.150	.233	–	–	–	–	–
Body mass index	.128	.327					
History of admission due to HF	.102	.420					
Hemoglobin	.249	.046	–	–	–	–	–
Log CRP	−.183	.165					
Albumin	.114	.365					
Concomitant respiratory infection	.026	.839					
Log length of bed rest	−.022	.862					
ROAG score	−.421	<.001	−.445	<.001	−3.017	0.805	−4.629 – −1.406
Barthel Index at admission	.080	.524					
Handgrip strength	.313	.087					
Gait speed for 10‐m walk	−.282	.124					
CONUT score	−.210	.098					
GNRI	.276	.034	–	–	–	–	–
Dietary energy intake	.329	.008	–	–	–	–	–
MMSE	.154	.810					

Abbreviations: CI, confidence interval; CRP, C‐reactive protein; CONUT, controlling nutritional status; GNRI, geriatric nutritional risk index; HF, heart failure; MMSE, mini‐mental state examination; ROAG, revised oral assessment guide.

## 4 DISCUSSION

The major findings of this study were as follows: (1) poor oral health, assessed using the ROAG score, is relatively common in elderly patients with acute HF; (2) patients with oral health problems had significantly more geriatric conditions, including decline in physical function, than those with normal oral health; and (3) the ROAG score was independently correlated with decline in the BI during hospitalization in elderly patients with acute HF.

In the current study, patients with oral health problems (ROAG score ≥9) was identified in 66.2% of the elderly patients with acute HF. Chronic inflammation caused by periodontal diseases is a risk factor for cardiac and cerebrovascular diseases, which are the major comorbidities of HF (Humphrey et al., 2008; Sen et al., 2018). General fatigue, dyspnea, delirium, and sleep disturbance accompanied by decompensated HF might contribute to reducing adherence to oral hygiene. In addition, dry mouth is often a consequence of polypharmacy, particularly as a side effect of cardiovascular agents (angiotensin‐converting enzyme inhibitors, beta‐blockers, and diuretics) (Kakudate et al., 2014; Smidt et al., 2011). However, oral health has been underrecognized in the assessment and management of patients with HF. Our data suggest the importance of oral assessment in the multidisciplinary management of elderly HF patients because poor oral health is highly prevalent and significantly associated with decline in physical function during hospitalization.

Our present study involving elderly patients with HF has shown that the ROAG score serves as a good predictor of changes in ADL, and its predictive ability is comparable to age, nutritional indices, and various other parameters. We have previously demonstrated that hospitalization for HF was significantly correlated with decreased BI as an assessment of ADL, and a decreased BI during hospitalization was associated with worse clinical outcomes (Uemura et al., 2018). It has also been reported that a decline in ADL due to acute HF is an independent risk factor of hospitalization for HF and mortality (Katano et al., 2021; Obata et al., 2021). Therefore, it is important to identify predictors of ADL decline during hospitalization in patients with HF. It has been demonstrated in elderly patients and patients with HF that age and the nutritional index are associated with ADL decline during hospitalization (Hsu et al., 2019; Takabayashi et al., 2019). Our data suggest that oral assessment of patients with HF provides additional information to prevent ADL decline during hospitalization by comprehensive care and rehabilitation to patients with poor oral health at an earlier stage.

In the present study, nutritional status, assessed by CONUT and GNRI, was worse in the poor oral health group than in the normal oral health group. Furthermore, patients with poor oral health had lower dietary energy intake. Previous studies have demonstrated that poor oral health is associated with periodontal disease, dental caries, hyposalivation, and tooth loss or edentulousness, which pose risks of chewing difficulties, decreased masticatory function, and dysphagia (Gil‐Montoya et al., 2015; Razak et al., 2014). These oral problems may induce a preference for soft and easily chewable food and a need for changes in food texture to prevent aspiration and choking, leading to poor nutritional intake and undernutrition, and finally to sarcopenia, frailty, and decreased physical function (Azzolino et al., 2019; Castrejón‐Pérez et al., 2012; Zenthoefer et al., 2014). Besides undernutrition, higher prevalence of cognitive impairment among patients with poor oral health might also be associated with physical deconditioning caused by prolonged bed rest or inactivity. Furthermore, a decline in physical and cognitive function would, in turn, reduce self‐care ability, including the ability to take care of one's oral health. Our data suggest that oral assessment of patients with HF using the ROAG score to screen for poor oral health may allow us to facilitate oral management to patients with poor oral health at an earlier stage. This may help improve nutritional status, enhance the patient self‐care ability, and prevent ADL decline during hospitalization for HF, leading to a better prognosis.

In the acute setting of decompensated HF, nurses have a pivotal role in oral assessment and care, because of the small number of dentists and oral hygienists. Several oral health assessment tools have been developed for non‐dental healthcare professionals (Andersson et al., 2002; Chalmers et al., 2005; Fjeld et al., 2017). Among these indices, the ROAG is not only a simple and comprehensive assessment but it also has favorable validity and reliability (Everaars et al., 2020). Oral assessment in patients with acute HF might have several influences on multidisciplinary disease management. First, patients who require consultation with in‐hospital or regional dental healthcare professionals are screened. Second, nurses receive feedback on oral care from their patients. Third, oral care is one of the fundamental self‐care activities after discharge, and thus, the information that an oral assessment provides is useful for educating patients on dental health compliance. Forth, several management strategies could be considered for patients with poor oral health. Nutritionists and dietitians could help to increase the dietary intake of the patients by changing meal content and food texture. Doctors and pharmacists could choose orally disintegrating tablets for the ease of taking medicines. Although the efficacy of multidisciplinary interventions for patients with poor oral health has not been fully elucidated, we believe that oral assessment could provide useful information for the multidisciplinary management of elderly patients with HF.

The present study had several limitations. First, this was a single‐center, retrospective study, and the sample size was relatively small. Second, the large number of eligible patients were excluded due to the lack of ROAG score. Oral health cannot be evaluated in patients in more critical conditions, and this could introduce bias. Future prospective studies are necessary with larger patient populations. Third, oral health was assessed by only one certified dysphagia nurse to avoid the interobserver variation. The results could not be generalized to the routine assessment by ward nurses.

## 5 CONCLUSION

Poor oral health, as assessed by the ROAG, is highly prevalent and oral assessment using the ROAG predicts a decline in physical function during hospitalization in elderly patients with acute HF. Thus, oral assessment during hospitalization could provide useful information to prevent ADL decline through comprehensive care, such as the facilitation of oral management, nutritional intervention, and rehabilitation in elderly patients with acute HF.

## AUTHOR CONTRIBUTIONS

Yusuke Uemura, Rei Shibata, Kenji Takemoto, and Shinji Ishikawa conceived the study, designed the protocol, contributed to data collection and preparation, analyzed all data, wrote the manuscript, and contributed to the interpretation of the results. Masato Watarai and Toyoaki Murohara were responsible for critical revision of the article for important intellectual content and approval of the final version.

## CONFLICT OF INTEREST STATEMENT

The authors declare no conflict of interest.

## Data Availability

The data that support the findings of this study are available from the corresponding author upon reasonable request.
